# Gemcitabine‐based conditioning compared to BEAM/BEAC conditioning prior to autologous stem cell transplantation for non‐Hodgkin lymphoma: No difference in outcomes

**DOI:** 10.1002/cam4.6965

**Published:** 2024-02-01

**Authors:** Huimin Liu, Hesong Zou, Dandan Shan, Wei Liu, Wenyang Huang, Weiwei Sui, Shuhui Deng, Tingyu Wang, Rui Lv, Mingwei Fu, Yan Xu, Shuhua Yi, Gang An, Yaozhong Zhao, Lugui Qiu, Dehui Zou

**Affiliations:** ^1^ State Key Laboratory of Experimental Hematology, National Clinical Research Center for Blood Diseases, Haihe Laboratory of Cell Ecosystem Institute of Hematology and Blood Diseases Hospital, Chinese Academy of Medical Sciences and Peking Union Medical College Tianjin China; ^2^ Tianjin Institutes of Health Science Tianjin China

**Keywords:** autologous stem cell transplantation, conditioning regimen, non‐Hodgkin lymphoma

## Abstract

**Background:**

High‐dose chemotherapy followed by autologous stem cell transplantation (ASCT) remains an effective treatment for non‐Hodgkin lymphoma (NHL). The limited availability of carmustine has prompted the exploration of novel alternative conditioning regimens. This study aimed to compare the efficacy and safety profile of GBM/GBC (gemcitabine, busulfan, and melphalan or cyclophosphamide) conditioning compared with the standard BEAM/BEAC regimens (carmustine, etoposide, cytarabine, and melphalan or cyclophosphamide) for ASCT in patients with NHL.

**Methods:**

A retrospective analysis was conducted on 231 NHL patients, who underwent ASCT from October 2010 to October 2021 at the Institute of Hematology & Blood Disease Hospital, including both first‐line and salvage settings. This resulted in the inclusion of 112 patients in the GBM/GBC arm and 92 in the BEAM/BEAC arm. Propensity score matching was employed to validate the results.

**Results:**

Disease subtype distribution was similar between the GBM/GBC and BEAM/BEAC groups, with diffuse large B‐cell lymphoma being the most common (58.9% vs. 58.7%), followed by PTCL (17.0% vs. 18.5%) and MCL (14.3% vs. 14.1%). At 3 months post‐ASCT, complete response (CR) rates were comparable (GBM/GBC 93.5% vs. BEAM/BEAC 91.1%; *p* = 0.607). The 4‐year progression‐free survival (78.4% vs. 82.3%; *p* = 0.455) and 4‐year overall survival (88.1% vs. 87.7%; *p* = 0.575) were also similar. Both groups exhibited low non‐relapse mortality at 4 years (GBM/GBC 1.8% vs. BEAM/BEAC 3.5%; *p* = 0.790) with no transplant‐related mortalities reported. The GBM/GBC cohort demonstrated a higher incidence of grade 3/4 oral mucositis and hepatic toxicity, whereas the BEAM/BEAC group had more frequent cases of bacteremia or sepsis (13 cases vs. 5 in GBM/GBC).

**Conclusions:**

The GBM/GBC regimen is effective and well‐tolerated, offering outcomes that are highly comparable to those in NHL patients conditioned with BEAM/BEAC, as demonstrated in a prognostically matched cohort.

## 1 INTRODUCTION

The introduction of novel targeted agents and chimeric antigen receptor (CAR) T‐cell therapies has significantly altered the therapeutic approach to lymphoma. Despite these advancements, high‐dose chemotherapy (HDT) followed by autologous stem cell transplantation (ASCT) continues to be the standard of care for patients with relapsed or refractory (r/r) lymphoma.^1^, ^2^, ^3^, ^4^ The role of HDT/ASCT as an initial consolidation therapy remains particularly effective, particularly in younger and fit patients with chemosensitive diseases such as mantle cell lymphoma (MCL), peripheral T‐cell lymphoma (PTCL), and diffuse large B‐cell lymphoma (DLBCL) that exhibit high‐risk features.^5^, ^6^, ^7^, ^8^

Historically, BEAM (BCNU, etoposide, cyclophosphamide, melphalan) has been the leading conditioning regimen for over 30 years, recognized for its established efficacy and safety.^9^, ^10^, ^11^, ^12^ Recent decades, however, have seen a limited availability of melphalan, leading to the emergence of alternative conditioning regimens, such as BEAC (which substitutes cyclophosphamide for melphalan), with reports indicating comparable toxicities and outcomes.^13^, ^14^, ^15^ After 2010, a shortage of BCNU prompted the exploration of alternatives like bendamustine or formustine, which, while clinically effective, are associated with new adverse effects, including renal injury.^9^, ^16^, ^17^ Additionally, MD Anderson Cancer Center developed a regimen comprising gemcitabine, busulfan, and melphalan (GBM), noted for its potent synergistic effect in inhibiting lymphoma cell line proliferation.^18^ Preliminary studies of the GBM regimen in r/r lymphomas have been encouraging,^19^ and a retrospective analysis revealed improved outcomes compared with BEAM in Hodgkin lymphoma (HL) patients.^20^

The limited availability of BCNU in China since 2017 led to our investigation into the potential of a modified conditioning regimen, GBM/GBC (gemcitabine, busulfan, and cyclophosphamide), for ASCT in patients with HL and non‐Hodgkin lymphoma (NHL) patients. Our initial results were promising, indicating high efficacy and good tolerability,^21^ highlighting the promise of GBM/GBC. However, the comparative efficacy and toxicity of these regimens, particularly in NHL patients who represent the majority of lymphoma cases, remain inadequately explored in both first‐line and salvage therapy contexts.

In light of these initial promising findings and the clear need for more comprehensive clinical data, we conducted a retrospective analysis of NHL patients within our institute's transplant cohort. Our study endeavors to provide valuable insights by comparing the efficacy and safety of GBM/GBC regimen against the established BEAM/BEAC regimens, employing propensity score matching (PSM) to enhance the robustness of our findings.

## 2 PATIENTS AND METHODS

### 2.1 Study design

**FIGURE 1:**
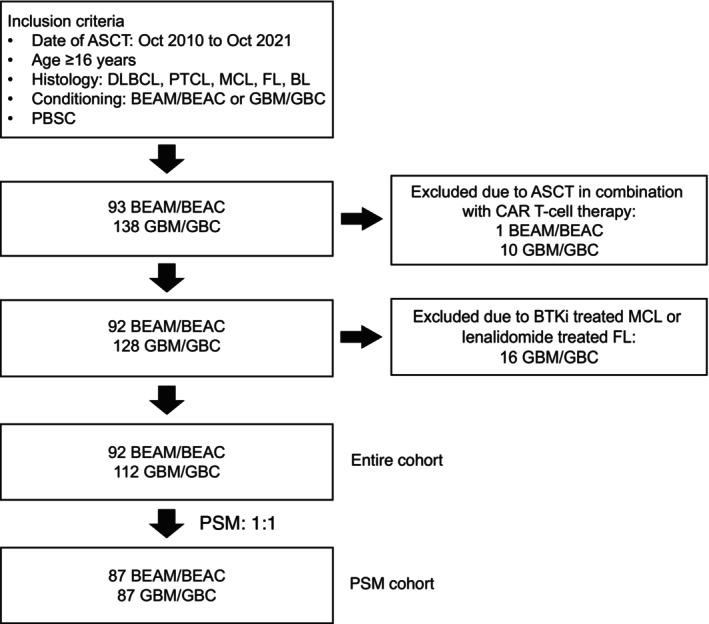
Flowchart of patient selection. ASCT, autologous stem cell transplantation; BEAM/BEAC, carmustine, etoposide, aracytin, melphalan/cyclophosphamide; BL, Burkitt's lymphoma; BTKi, Bruton tyrosine kinase inhibitor; CAR‐T, chimeric antigen receptor T‐cell immunotherapy; DLBCL, diffuse large B‐cell lymphoma; FL, follicular lymphoma; GBMGBC, gemcitabine, busulfan, melphalan/cyclophosphamide; MCL, mantle cell lymphoma; PTCL, peripheral T‐cell lymphoma.

We retrospectively analyzed data from 231 consecutive NHL patients who received ASCT from October 2010 to October 2021 at the Institute of Hematology & Blood Disease Hospital, Chinese Academy of Medical Sciences & Peking Union Medical College. Eligibility criteria for inclusion were: A diagnosis of DLBCL, PTCL, MCL, follicular lymphoma (FL), or Burkitt's lymphoma (BL); absence of prior stem cell transplantation; conditioning with either GBM/GBC or BEAM/BEAC regimens; and the use of peripheral blood as a stem cell source. To mitigate potential bias, we excluded patients who underwent ASCT followed by CAR T‐cell therapy, MCL patients treated with Bruton's tyrosine kinase inhibitors (BTKi), and FL patients treated with lenalidomide (Figure 1). The study was approved by the Ethics Committee and Human Research Committee of the Institute of Hematology & Blood Disease Hospital. Clinical data were collected from the institution's bone marrow and peripheral blood stem cell data registry. All patients provided written informed consent prior to transplantation.

### 2.2 Conditioning regimens and supportive treatment

GBM/GBC involved the administration of gemcitabine (loading bolus of 75 mg/m^2^ followed by continuous infusion of 1800 mg/m^2^ over 3 h on Days −7 and − 3), busulfan (105 mg/m^2^, Day −7 to Day −5), and either melphalan (60 mg/m^2^, Day −3 to Day −2) or cyclophosphamide (50 mg/kg, Day −3 to Day −2). The BEAM/BEAC regimens included carmustine (300 mg/m^2^ on Day −6), etoposide (200 mg/m^2^, Day −5 to Day −2), cytarabine (400 mg/m^2^, Day −5 to Day −2), and either melphalan (140 mg/m^2^ on Day −1) or cyclophosphamide (30 mg/kg, Day −5 to Day −2). Peripheral autologous stem cells were reintroduced on Day 0. Following reinfusion, pegylated recombinant human granulocyte colony‐stimulating factor was administered within 48–72 h, or alternatively, recombinant human granulocyte colony‐stimulating factor (5 μg/kg/day) was administered on Day 5 and was continued until the leukocyte counts stabilized at a minimum of 1.0 × 10^9^/L for three consecutive days. Routine supportive care included prophylactic administration of acyclovir, compounded sulfamethoxazole, and ursodiol according to a predefined schedule.

### 2.3 Measurements and definitions

The response status was evaluated using positron emission tomography (PET) and/or computed tomography (CT), following the Lugano criteria.^22^ Prior to ASCT and 3 months afterwards, all patients underwent evaluation. Neutrophil engraftment was defined as achieving a sustained absolute neutrophil count above 0.5 × 10^9^/L for three consecutive days. Platelet engraftment was defined as a platelet count exceeding 20 × 10^9^/L without the need for platelet transfusions over a period of at least seven consecutive days. Transplant‐related toxicities were evaluated using the NCI Common Terminology Criteria for Adverse Events (CTCAE v5.0). Progression‐free survival (PFS) was defined as the period from stem cell infusion to either disease progression or relapse, any cause of death, or the last follow‐up. Overall survival (OS) was calculated from the date of transplant to the date of death from any cause or the last follow‐up. The cumulative incidence of relapse or progression (CIR) and non‐relapse/progression mortality (NRM) were also measured from the time of stem cell infusion to disease progression or death.

### 2.4 Statistical analysis

Patients undergoing conditioning with GBM/GBC were matched with those receiving BEAM/BEAC, based on several factors: age, sex, type of NHL, previous therapy lines, and pre‐ASCT response. A PSM was employed using 1:1 nearest neighbor matching. For continuous variables, comparisons were made using the two‐sample *t*‐test or the Mann–Whitney *U*‐test. Categorical variables were analyzed using the chi‐squared test or Fisher's exact test. PFS and OS probabilities were calculated using the Kaplan–Meier method. The CIR and NRM were estimated using a competing risk model (Gray's test). To identify factors independently correlating with PFS or OS, multivariate analyses were performed using the Cox proportional hazards model. All statistical analyses were conducted using SPSS (version 27.0) and R version 4.2.1, with statistical significance defined as a *p* < 0.05.

## 3 RESULTS

### 3.1 Baseline characteristics

Table 1 presents the clinical characteristics of 231 patients with NHL, who underwent ASCT. Exclusions included 27 patients receiving ASCT followed by CAR T‐cell therapy or treated for FL/MCL with new drugs (Figure 1). In the final analysis, 112 patients conditioned with GBM/GBC and 92 with BEAM/BEAC were evaluated to compare outcomes. Baseline characteristics were generally balanced across the entire cohort (Table 1) and the PSM cohort (Table S1). The GBM/GBC regimens were mainly used from 2018 to 2021, whereas BEAM/BEAC regimens were more common during 2010–2017. Median ages were 43 in the GBM/GBC group and 39 in the BEAM/BEAC group (*p* = 0.364). Disease subtype distribution was similar in both groups, with DLBCL being the most prevalent (GBM/GBC 58.9% vs. BEAM/BEAC 58.7%), followed by PTCL (17.0% vs. 18.5%) and MCL (14.3% vs. 14.1%). Disease burden at diagnosis was comparable: advanced stages in 80.4% and 77.2% of patients, approximately half with elevated lactate dehydrogenase (LDH), and one‐third with bone marrow involvement. TP53 abnormalities were more common in the GBM group (22.5% vs. 10.5%), but this was not statistically different (*p* = 0.212). There were no significant differences in rates of refractoriness to initial treatment, prior lines of therapy, and response status before ASCT. Our cohort exhibited a higher incidence of first‐line transplantation compared with historical reports. Among patients with DLBCL, most exhibited high‐risk clinical features such as an age‐adjusted International Prognostic Index (aaIPI) of 2–3 and positive interim PET scan results after induction treatment, or biological high‐risk features including double expression, double‐/triple‐hit, and *TP53* alterations (Table S2).

**TABLE 1:** Patient baseline characteristics before propensity score matching (PSM).

Variable	Overall (*n* = 231)	GBM/GBC (*n* = 112)	BEAM/BEAC (*n* = 92)	*p*‐value
Median age (range)	42 (16–65)	43 (18–64)	39 (18–65)	0.364
Sex				0.417
Male	130 (56.3)	66 (58.9)	49 (53.3)	
Female	101 (43.7)	46 (41.1)	43 (46.7)	
Histology				0.988
DLBCL	130 (56.3)	66 (58.9)	54 (58.7)	
PTCL	36 (15.5)	19 (17.0)	17 (18.5)	
MCL	35 (15.2)	16 (14.3)	13 (14.1)	
FL	24 (10.4)	8 (7.1)	5 (5.4)	
BL	6 (2.6)	3 (2.7)	3 (3.3)	
ECOG PS				0.778
0–1	193 (83.5)	93 (83.0)	75 (81.5)	
2–4	38 (16.5)	19 (17.0)	17 (18.5)	
Ann Arbor stage				0.579
I–II	44 (19.0)	22 (19.6)	21 (22.8)	
III–IV	187 (81.0)	90 (80.4)	71 (77.2)	
Elevated LDH	109 (47.2)	56 (50.9)	43 (50.0)	0.899
Extra‐nodal involvement	159 68.8	76 (67.9)	63 (68.5)	0.925
BMI	80 (34.6)	33 (29.7)	30 (32.6)	0.659
TP53 alterations	28/153 (18.3)	20/93 (21.5)	4/37 (10.8)	0.212
Prior lines of therapy				0.968
1	161 (69.7)	81 (72.3)	64 (69.6)	
≥ 2	70 (30.3)	31 (27.7)	28 (30.4)	
Primary refractory	34 (14.7)	12 (10.7)	14 (15.2)	0.337
Response pre‐ASCT				0.989
CR	177 (76.6)	90 (80.4)	74 (80.4)	
CR1	136 (58.9)	68 (60.7)	56 (60.9)	
PR	50 (21.7)	22 (19.6)	18 (19.6)	
PR1	25 (10.8)	13 (11.6)	8 (8.7)	
SD/PD	4 (1.7)	0	0	
Year of ASCT				<0.001*
2010–2017	99 (42.9)	12 (10.7)	87 (94.6)	
2018–2021	132 (57.1)	100 (89.3)	12 (10.7)	

Abbreviations: ASCT, autologous stem cell transplantation; BEAM/BEAC, carmustine, etoposide, cytarabine, melphalan/cyclophosphamide; BL, Burkitt's lymphoma; BMI, bone marrow involvement; CR, complete remission; CR1, complete remission in first‐line treatment; DLBCL, diffuse large B‐cell lymphoma; ECOG PS, Eastern Cooperative Oncology Group Performance Status; FL, follicular lymphoma; GBM/GBC, gemcitabine, busulfan, melphalan/cyclophosphamide; LDH, lactate dehydrogenase; MCL, mantle cell lymphoma; PD, progressive disease; PR, partial remission; PR1, partial remission in first‐line treatment; PTCL, peripheral T‐cell lymphoma; SD, stable disease.

* Statistical significance *p* < 0.05.

### 3.2 Engraftment and toxicity

Table 2 presents the engraftment and toxicity data for the entire cohort. In the GBM/GBC and BEAM/BEAC groups, the median doses of transplanted CD34+ cells were 2.92 × 10^6^/kg (range, 1.12–21.33) and 3.02 × 10^6^/kg (range, 1.53–18.00), respectively (*p* = 0.805). Neutrophil engraftment occurred at a median of 10 days (*p* = 0.331) in both groups, while the median times for platelet engraftment were 12 and 11 days in each group, respectively (*p* = 0.130). No instances of graft failure were observed in the study.

**TABLE 2:** Engraftment and toxicities in patients before propensity score matching (PSM).

Variable	GBM/GBC (*n* = 112)	BEAM/BEAC (*n* = 92)	*p*‐value
Engraftment			
CD34+ reinfused (×10^6^/kg)	2.92 (1.12–21.33)	3.02 (1.53–18.00)	0.805
Days to ANC >0.5 × 10^9^/L	10 (8–28)	10 (8–21)	0.331
Days to platelet >20 × 10^9^/L	12 (0–63)	11 (0–38)	0.130
*Toxicity*			
Hematologic toxicity, grade 3/4			
Granulocytopenia	112 (100)	92 (100)	1.000
Anemia	92 (82.1)	76 (82.6)	0.931
Thrombocytopenia	112 (100)	92 (100)	1.000
Hemorrhage	3 (2.7)	0	0.254
Febrile neutropenia	106 (94.6)	82 (89.1)	0.145
Documented infection			
Bacterial	16 (14.3)	23 (25.0)	0.053
Viral	4 (3.6)	0	0.129
Fungal	6 (5.4)	3 (3.3)	0.517
CMV	2 (1.8)	0	0.502
Non‐hematologic toxicity, grade 3/4			
Oral mucositis	41 (36.6)	13 (14.1)	<0.001*
Diarrhea	12 (10.7)	12 (13.0)	0.607
Nausea/vomiting	22 (19.6)	19 (20.7)	0.858
Skin rash	2 (1.8)	0 (0)	0.502
Hepatic toxicity	32 (28.2)	3 (3.3)	<0.001*
Cardiac toxicity	0	0	
Renal toxicity	0	0	
Neurological toxicity	0	0	
VOD	0	0	
TRM	0	0	

Abbreviations: ANC, absolute neutrophil count; BEAM/BEAC, carmustine, etoposide, cytarabine, melphalan/cyclophosphamide; CMV, cytomegalovirus; GBM/GBC, gemcitabine, busulfan, melphalan/cyclophosphamide; TRM, transplant‐related mortality; VOD, veno‐occlusive disease.

* Statistical significance *p* < 0.05.

Grade 3/4 hematologic toxicities, including neutropenia, thrombocytopenia, and anemia, were observed in nearly all patients, without significant differences between the GBM/GBC and BEAM/BEAC groups. Febrile neutropenia was also prevalent in the majority of both groups (94.6% vs. 89.1%, *p* = 0.145). Notably, bacterial infections were more frequent in the BEAM/BEAC group compared with the GBM/GBC group (14.3% vs. 25.0%, *p* = 0.053), with a higher incidence of bacteremia or sepsis in the BEAM/BEAC group (*n* = 13) than in the GBM/GBC group (*n* = 5). The occurrence of grade 3/4 non‐hematologic toxicities, such as diarrhea, nausea/vomiting, skin rash, cardiotoxicity, renal toxicity, and neurotoxicity, was comparable between the two groups. However, a higher incidence of grade 3/4 oral mucositis (36.6% vs. 14.1%; *p* < 0.001) and hepatic toxicity (28.2% vs. 3.3%; *p* < 0.001) was noted in the GBM/GBC group compared with the BEAM/BEAC group. These adverse events demonstrated rapid resolution following appropriate interventions. There were no reports of veno‐occlusive disease, and transplant‐related mortality (TRM) was absent at 3 months post‐ASCT in both groups. The engraftment and toxicities in the PSM‐matched cohorts aligned closely with those observed in the entire cohort (Table S3).

### 3.3 Outcomes

**FIGURE 2:**
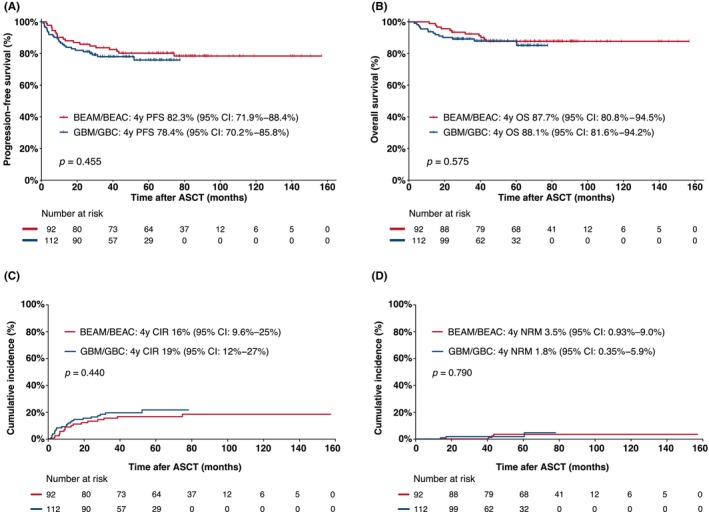
Treatment outcomes after transplantation among patients in the BEAM/BEAC and GBM/GBC groups of the entire cohort. (A) Kaplan–Meier estimate curves of progression‐free survival (PFS) rates; (B) Kaplan–Meier estimate curves of overall (OS) rates; (C) Cumulative incidence of relapse or progression (CIR); (D) Incidence of non‐relapse mortality (NRM). BEAM/BEAC, carmustine, etoposide, aracytin, melphalan/cyclophosphamide; GBM/GBC, gemcitabine, busulfan, melphalan/cyclophosphamide.

The median follow‐up duration was 46.6 months (range, 3.0–77.6) for the GBM/GBC group and 80.7 months (range, 11.4–156.8) for the BEAM/BEAC cohort in the entire study population. At 3 months post‐ASCT, the complete response (CR) rates were 93.5% in the GBM/GBC group and 91.1% in the BEAM/BEAC group (*p* = 0.607). The PFS rates at 4 years were 78.4% (95% CI: 70.2%–85.8%) for the GBM/GBC group and 82.3% (95% CI: 71.9%–88.4%) for the BEAM/BEAC arm (*p* = 0.455) (Figure 2A). The 4‐year OS rates were 88.1% (95% CI: 81.6%–94.2%) for the GBM/GBC group and 87.7% (95% CI: 80.8%–94.5%) for the BEAM/BEAC group (*p* = 0.575) (Figure 2B). No significant differences were observed in the 4‐year PFS and OS rates between the two groups across the major disease subtypes (Figure S1). Notably, patients in the frontline transplantation arm exhibited higher 4‐year PFS rates (82.9% vs. 68.6%; *p* = 0.012) compared with those in the non‐frontline transplantation arm. A marginally higher 4‐year OS rate was also noted in the frontline group, although it did not reach statistical significance (90.2% vs. 80.2%; *p* = 0.053) (Figure S2A,B).

The CIR (GBM/GBC: 19.0% vs. BEAM/BEAC: 16.0%; *p* = 0.440) and NRM (GBM/GBC: 1.8% vs. BEAM/BEAC: 3.5%; *p* = 0.790) at 4 years did not differ between the two groups (Figure 2C,D). In the GBM/GBC cohort, causes of death included 11 patients from disease progression, one from hepatic failure at 16.8 months posttransplant (with a prior history of hepatitis B virus), one from infection following salvage CAR‐T therapy, and one from COVID‐19 at 60.3 months posttransplant. In the BEAM/BEAC cohort, eight patients (9.2%) died from disease progression, two (1.1%) from second primary malignancies (acute myeloid leukemia or myelodysplastic syndromes) at 36 months post‐ASCT, and one (1.1%) in a traffic accident at 43 months post‐ASCT.

**FIGURE 3:**
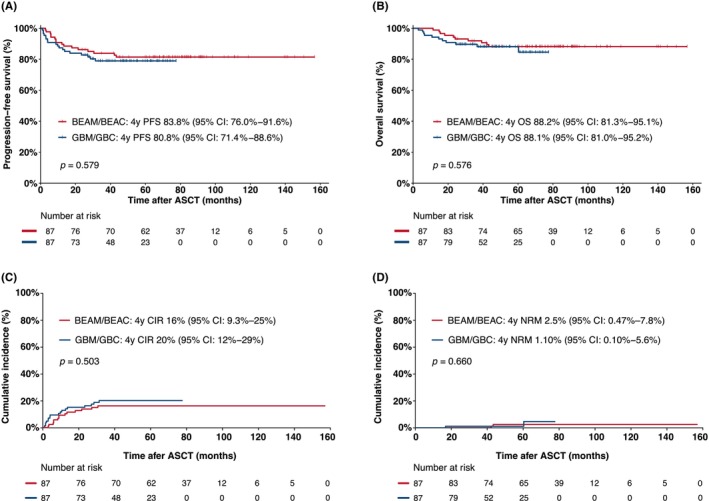
Treatment outcomes after transplantation among patients in the BEAM/BEAC and GBM/GBC groups of the PSM cohort. (A) Kaplan–Meier estimate curves of progression‐free survival (PFS) rates; (B) Kaplan–Meier estimate curves of overall (OS) rates; (C) Cumulative incidence of relapse or progression (CIR); (D) Incidence of non‐relapse mortality (NRM). BEAM/BEAC, carmustine, etoposide, aracytin, melphalan/cyclophosphamide; GBM/GBC, gemcitabine, busulfan, melphalan/cyclophosphamide; PSM, propensity score matching.

To validate these findings, a parallel analysis was conducted in the PSM cohort. This analysis revealed no significant differences in 4‐year PFS, OS, CIR, and NRM between the GBM/GBC and BEAM/BEAC groups, as depicted in Figure 3.

**FIGURE 4:**
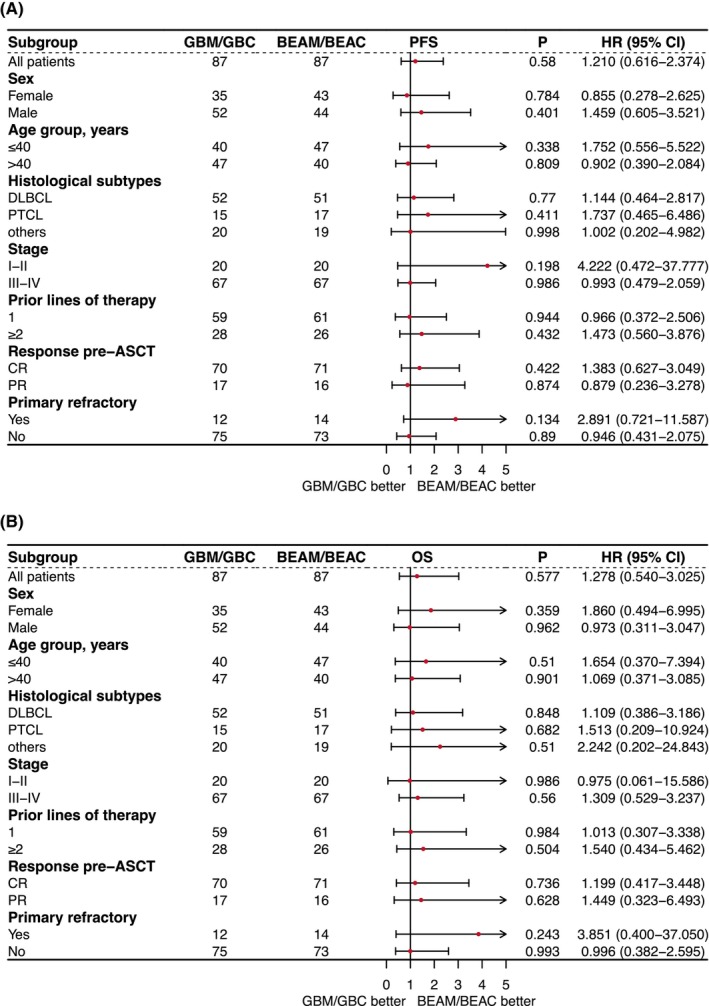
Forest plot of subgroups analysis on progression‐free survival (PFS) and overall survival (OS) in the PSM cohort by conditioning regimens. ASCT, autologous stem cell transplantation; BEAM/BEAC, carmustine, etoposide, aracytin, melphalan/cyclophosphamide; CR, complete response; DLBCL, diffuse large B‐cell lymphoma; GBM/GBC, gemcitabine, busulfan, melphalan/cyclophosphamide; MCL, mantle cell lymphoma; PR, partial response; PSM, propensity score matching; PTCL, peripheral T‐cell lymphoma.

Figure 4 shows the comparison of PFS in the PSM cohort by conditioning regimens across subgroups defined by standard clinical features, including age, sex, histological subtypes, stages, prior lines of therapy, response pre‐ASCT, and primary refractory status. This comparison did not show significant differences in PFS or OS between arms for any subgroups. These consistent results were also observed in the entire cohort, as illustrated in Figure S3.

### 3.4 Salvage treatment for relapse/progression post‐ASCT

Among the 40 patients with disease progression, 25 (62.5%) underwent salvage treatment. This included 16 in the GBM/GBC group and 9 in the BEAM/BEAC group (Table 3). Seven patients in the GBM group and one patient in the BEAM group were treated with CD19 CAR T‐cell therapy. Of these, six patients achieved CR and two patients achieved PR. Additionally, one patient from each group underwent allogeneic hematopoietic stem cell transplantation, both achieving CR lasting over a year. Furthermore, 13 patients received immunochemotherapy with novel agents including lenalidomide, BTK inhibitors, chidamide, and anti‐PD1 antibodies. Within this subgroup, eight patients achieved CR and one patient achieved PR.

**TABLE 3:** Salvage treatments following autologous stem cell transplantation (ASCT).

Treatment	GBM/GBC	BEAM/BEAC
CAR T‐cell therapy	7 (5 CR, 2 PR)	1 (CR)
Allo–SCT	1 (CR)	1 (CR)
Chidamide–based	3 (2 CR, 1 PD)	1 (PD)
Lenalidomide‐based	1 (PD)	3 (2 CR, 1 PD)
BTKi–based	2 (2 CR)	2 (2 CR)
Anti–PD1–based	1 (PR)	0
Brentuximab vedotin	1 (CR)	0
BR	0	1 (CR)
Palliative therapy	6	6
Unknown	1	2

Abbreviations: Allo‐SCT, allogeneic hematopoietic stem cell transplantation; BEAM/BEAC, carmustine, etoposide, cytarabine, melphalan/cyclophosphamide; BR, bendamustine plus rituximab; BTKi, Bruton tyrosine kinases inhibitor; CAR, chimeric antigen receptor; CR, complete response; GBM/GBC, gemcitabine, busulfan, melphalan/cyclophosphamide; PD, progressive disease; PR, partial response.

## 4 DISCUSSION

In the present study, we examined long‐term survival outcomes and toxicity profiles in a large cohort comparing GBM/GBC and BEAM/BEAC conditioning regimens, followed by ASCT for patients with NHL in their first CR/PR or those with r/r NHL. Across the entire cohort, there were no significant differences in 4‐year PFS (78.4% vs. 82.3%) and OS (88.1% vs. 87.7%) between GBM/GBC and BEAM/BEAC arms. To minimize potential imbalances between the two groups, we utilized the PSM method, confirming similar survival outcomes.

Cyclophosphamide alternative regimens have gained prominence following a shortage of melphalan. Some studies have suggested that BEAC may increase the risk of cardiotoxicity^23^, ^24^; however, a recent large‐scale retrospective matched cohort study by the European Society for Blood and Marrow Transplantation (EBMT) found comparable outcomes and toxicity profiles for ASCT conditioned with BEAM or BEAC.^15^ In line with previous literature,^9^, ^15^, ^24^ the most prevalent grade 3/4 extra‐hematologic toxicities in our BEAM/BEAC group were nausea/vomiting (20.7%), mucositis (14.1%), and diarrhea (13.0%). Regarding the GBM regimen, Nieto et al. reported that the most common ≥grade 3 adverse events in r/r lymphoma and multiple myeloma were mucositis (27%–41%), dermatitis (7%–10%), hyperbilirubinemia (12%–19%), and elevated transaminases (9%–19%).^19^, ^25^, ^26^ To improve tolerance among Asians, we modified the gemcitabine dosage to 1800 mg/m^2^ for 2 days and busulfan to 105 mg/m^2^ for 3 days in our GBM/GBC cohort. As a result, we observed a lower incidence of severe skin rash (1.8%), with mucositis (36.6%) as the most prevalent adverse event, followed by hepatic toxicities (28.2%).

In the comparative analysis of treatment‐related complications among our study groups, we observed no occurrences of TRM or veno‐occlusive disease. The incidence of common adverse events such as hematologic toxicities, nausea/vomiting, diarrhea, and skin rash showed no statistical difference between the groups. Notably, bacterial infections were more common in the BEAM/BEAC group compared with the GBM/GBC group (25.0% vs. 14.3%, *p* = 0.053), with a higher incidence of bacteremia or sepsis in the BEAM/BEAC group (*n* = 13) than in the GBM/GBC group (*n* = 5). Furthermore, the GBM/GBC group exhibited a higher incidence of oral mucositis (36.6% vs. 14.1%) and hepatic toxicity (28.2% vs. 3.3%) compared with the BEAM/BEAC group. However, these adverse events were rapidly manageable within 1 week through appropriate interventions. The low incidence of mucositis in the BEAM/BEAC group might be attributed to 70.1% of patients undergoing ASCT in their first remission and the administration of BEAC in some patients, which aligns with previous reports.^14^, ^15^ Considering that busulfan is a component of the BEAM/BEAC regimens, we hypothesized that the elevated occurrence of oral mucositis in GBM/GBC regimens might be more strongly associated with gemcitabine use. Despite over half of our cohort substituting melphalan with cyclophosphamide, it is noteworthy that no cardiac toxicity‐related mortalities were observed. These findings collectively suggest that GBM/GBC is safe for NHL patients.

In our series, we noted that the CR rates in the GBM/GBC and BEAM/BEAC groups increased from 80.4% and 80.4% (*p* = 0.989) before transplantation to 93.5% and 91.1% (*p* = 0.607) at 3 months posttransplantation, respectively. This observation suggests that ASCT may further enhance the depth of remission in NHL patients. There was no significant difference in the 4‐year PFS (78.4% vs. 82.3%; *p* = 0.455) and 4‐year OS (88.1% vs. 87.7%; *p* = 0.575). The 4‐year NRM rates were 1.8% and 3.5% (*p* = 0.790) in the GBM/GBC and BEAM/BEAC arms, respectively. Only two studies have conducted a comparative analysis of GBM and BEAM. In Nieto et al.'s study on a HL cohort,^26^ the authors reported a higher 2‐year PFS rate for GBM compared with BEAM (65% vs. 51%; *p* = 0.008) and an improved OS (89% vs. 73%; *p* = 0.0003). Similarly, a recent study by Alkhaldi et al.^27^ demonstrated better 5‐year OS rates with R‐vorinostat/GBM compared with R‐BEAM (82% vs. 65%), even though patients with r/r primary mediastinal large B‐cell lymphoma had undergone more prior lines of therapy. Considering the frequent use of gemcitabine in conjunction with other agents for second‐line salvage therapy in r/r lymphoma,^28^, ^29^ we postulated that the favorable outcomes in these studies might be attributed to the efficacy of gemcitabine in r/r patients. The majority of patients in our study, however, received first‐line consolidation transplants while in remission status. Additionally, the GBM arm in Alkhaldi et al.'s study received concurrent treatment with vorinostat, which may have contributed to an enhanced antitumor effect.

Posttransplant salvage therapy has historically presented significant challenges. Recent advances in small‐molecule inhibitors and CAR T‐cell therapy, however, have introduced new prospects. Several recent substantial clinical studies have highlighted the impressive response rates of CAR T‐cell therapy in NHL patients, even after multiple prior lines of therapy.^30^, ^31^, ^32^, ^33^ In our cohort of 40 patients experiencing posttransplant disease progression, 25 underwent salvage therapy. This treatment led to encouraging results, with 21 patients achieving at least a PR. Notably, eight patients underwent CD19 CAR T‐cell therapy, resulting in five patients achieving CR and two achieving PR.

Our study has several limitations. First, its retrospective design poses inherent constraints. Although we used PSM to equilibrate baseline characteristics between the two groups, with no statistical differences in most variables including prior therapies, a prospective study is needed to corroborate these findings on efficacy. Second, the study's span over an 11‐year period introduces potential bias linked to evolving treatments or enhanced supportive care, such as more effective antibiotics. It is pertinent to acknowledge that we excluded patients receiving novel drugs or CAR T‐cell therapy. Notably, TRM in both groups was markedly low, suggesting that the temporal scope of the study is unlikely the predominant factor influencing the enhanced outcomes.

In conclusion, our study demonstrates that the modified GBM/GBC conditioning regimens for ASCT are effective and well‐tolerated. These regimens offer a promising alternative to the traditional BEAM/BEAC conditioning regimens in the treatment of Asian patients with NHL.

## AUTHOR CONTRIBUTIONS

**Huimin Liu:** Conceptualization (equal); formal analysis (equal); methodology (equal); writing – original draft (equal). **Hesong Zou:** Data curation (equal); formal analysis (equal); methodology (equal); writing – original draft (equal). **Dandan Shan:** Data curation (equal). **Wei Liu:** Resources (equal). **Wenyang Huang:** Resources (equal). **Weiwei Sui:** Resources (equal). **Shuhui Deng:** Resources (equal). **Tingyu Wang:** Resources (equal). **Rui Lv:** Resources (equal). **Mingwei Fu:** Resources (equal). **Yan Xu:** Resources (equal). **Shuhua Yi:** Resources (equal). **Gang An:** Resources (equal). **Yaozhong Zhao:** Resources (equal). **Lugui Qiu:** Conceptualization (equal); funding acquisition (equal); supervision (equal); writing – review and editing (equal). **Dehui Zou:** Conceptualization (equal); project administration (lead); supervision (equal); writing – review and editing (equal).

## FUNDING INFORMATION

This work was supported by the CAMS Innovation Fund for Medical Sciences (CIFMS 2022‐I2M‐1‐022).

## CONFLICT OF INTEREST STATEMENT

The authors declare no competing interests.

## CONSENT STATEMENT

The study was approved by the Ethics Committee of the Institute of Hematology & Blood Disease Hospital, Chinese Academy of Medical Sciences and Peking Union Medical College. Informed consent was obtained from all patients included in the study.

## Supporting information


**Data S1:** Supporting information.

## Data Availability

The data used in this study are available upon request from the corresponding author.
