# Supplementary material for: Gemcitabine‐based conditioning compared to BEAM/BEAC conditioning prior to autologous stem cell transplantation for non‐Hodgkin lymphoma: No difference in outcomes
Source: Cancer Med. 2024 Feb 1;13(2):e6965. doi: 10.1002/cam4.6965 (PMC10831922; doi:10.1002/cam4.6965)

**Supplementary Table 1. Patients’ baseline characteristics after PSM.**

| Variable | GBM/GBC  (n=87) | BEAM/BEAC (n=87) | p-value |
| --- | --- | --- | --- |
| Median age(range) | 42 (17-62) | 39 (16-65) | 0.367 |
| Sex |  |  | 0.387 |
| Male | 52 (59.8) | 44 (50.6) |  |
| Female | 35 (40.2) | 43 (49.4) |  |
| Histology |  |  | 0.965 |
| DLBCL | 52 (59.8) | 51 (58.6) |  |
| PTCL | 15 (17.2) | 17 (19.5) |  |
| MCL | 11 (12.6) | 12 (13.8) |  |
| FL | 6 (6.9) | 4 (4.6) |  |
| BL | 3 (3.4) | 3 (3.4) |  |
| ECOG PS |  |  | 1.000 |
| 0-1 | 72 (82.8) | 71 (81.6) |  |
| 2-4 | 15 (17.2) | 16 (18.4) |  |
| Ann Arbor stage |  |  | 1.000 |
| I-II | 20 (23.0) | 20 (23.0) |  |
| III-IV | 67 (77.0) | 67 (77.0) |  |
| Elevated LDH | 43 (50.6) | 41 (50.6) | 1.000 |
| Extra-nodal involvement | 59 (67.8) | 59 (67.8) | 1.000 |
| BMI | 24 (27.9) | 26 (29.9) | 0.905 |
| TP53 alterations | 16/71 (22.5) | 3/33 (9.1) | 0.168 |
| Prior lines of therapy |  |  | 0.938 |
| 1 | 59 (67.8) | 61 (70.1) |  |
| ≥ 2 | 28 (32.2) | 26 (29.9) |  |
| Primary refractory | 12 (13.8) | 14 (16.1) | 0.832 |
| Response pre–ASCT |  |  | 0.681 |
| CR | 70 (80.5) | 71 (81.6) |  |
| CR1 | 50 (57.5) | 55 (63.2) |  |
| PR | 17 (19.5) | 16 (18.4) |  |
| PR1 | 9 (10.3) | 6 (6.9) |  |
| SD/PD | 0 | 0 |  |
| Year of ASCT |  |  | <0.001* |
| 2010–2017 | 9 (10.3) | 83 (95.4) |  |
| 2018–2021 | 78 (89.7) | 4 (4.6) |  |

PSM, propensity score matching; GBM/GBC, gemcitabine, busulfan, melphalan/cyclophosphamide; BEAM/BEAC, carmustine, etoposide, cytarabine, melphalan/cyclophosphamide; DLBCL, diffuse large B-cell lymphoma; PTCL, peripheral T-cell lymphoma; MCL, mantle cell lymphoma; FL, follicular lymphoma; BL, Burkitt’s lymphoma; ECOG PS, Eastern Cooperative Oncology Group Performance Status; LDH, lactate dehydrogenase; BMI, bone marrow involvement; ASCT, autologous stem cell transplantation; CR, complete remission; CR1, complete remission in first-line treatment; PR, partial remission; PR1, partial remission in first-line treatment; SD, stable disease; PD, progressive disease. *Statistical significance *P*< 0.05.

**Supplementary Table 2. Distribution of different disease subtypes that underwent front-line transplantation in the entire cohort.**

| Histology | GBM/GBC (n=81) | BEAM/BEAC (n=64) |
| --- | --- | --- |
| DLBCL | 45/66 (68.2) | 33/54 (61.1) |
| aaIPI ≥2 | 26/45 (57.8) | 14/33 (42.4) |
| EN sites involvement ≥2 | 29/45 (64.4) | 13/33 (39.4) |
| DE | 14/43 (32.6) | 12/28 (42.9) |
| TP53 alterations | 12/41 (29.3) | 0/12 (0) |
| PTCL | 14/19 (73.7) | 12/17 (70.6) |
| MCL | 16/16 (100.0) | 13/13 (100.0) |
| FL | 4/8 (50.0) | 3/5 (60.0) |
| BL | 2/3 (66.7) | 3/3 (100.0) |

GBM/GBC, gemcitabine, busulfan, melphalan/cyclophosphamide; GBC, gemcitabine, busulfan, cyclophosphamide; BEAM/BEAC, carmustine, etoposide, cytarabine, melphalan/cyclophosphamide; DLBCL, diffuse large B-cell lymphoma; PTCL, peripheral T-cell lymphoma; MCL, mantle cell lymphoma; FL, follicular lymphoma; BL, Burkitt’s lymphoma; aaIPI, age-adjusted international prognostic index; EN, extra-nodal; DE, double expressor.

**Supplementary Table 3. Engraftment and toxicities in patients after PSM.**

| Variable | GBM/GBC (n=87) | BEAM/BEAC (n=87) | p-value |
| --- | --- | --- | --- |
| Engraftment |  |  |  |
| CD34+ reinfused (×10^6^/kg) | 3.15 (1.12-21.33) | 4.02 (1.53-18.00) | 0.728 |
| Days to ANC > 0.5 × 10^9^/L | 10 (8-28) | 10 (8-21) | 0.653 |
| Days to platelet > 20 × 10^9^/L | 11 (0-63) | 11 (0-38) | 0.235 |
| Toxicity |  |  |  |
| Hematologic toxicity, grade 3/4 |  |  |  |
| Granulocytopenia | 87 (100) | 87 (100) | 1.000 |
| Anemia | 70 (80.5) | 72 (82.8) | 0.845 |
| Thrombocytopenia | 87 (100) | 87 (100) | 1.000 |
| Hemorrhage | 3 (3.4) | 0 | 0.254 |
| Febrile neutropenia | 82 (94.3) | 77 (88.5) | 0.280 |
| Documented infection |  |  |  |
| Bacterial | 10 (11.5) | 20 (23.0) | 0.071 |
| Viral | 3 (3.4) | 0 (0.0) | 0.244 |
| Fungal | 6 (6.9) | 3 (3.3) | 0.517 |
| CMV | 1 (1.1) | 0 (0.0) | 1.000 |
| Non-hematologic toxicity, grade 3/4 |  |  |  |
| Oral mucositis | 31 (35.6) | 11 (12.6) | <0.001* |
| Diarrhea | 9 (10.3) | 12 (13.8) | 0.643 |
| Nausea/vomiting | 13 (14.9) | 18 (20.7) | 0.428 |
| Skin rash | 2 (2.3) | 0 (0) | 0.477 |
| Hepatic toxicity | 24 (27.6) | 3 (3.3) | <0.001* |
| Cardiac toxicity | 0 | 0 |  |
| Renal toxicity | 0 | 0 |  |
| Neurological toxicity | 0 | 0 |  |
| VOD | 0 | 0 | 1.000 |
| TRM | 0 | 0 | 1.000 |

GBM/GBC, gemcitabine, busulfan, melphalan/cyclophosphamide; BEAM/BEAC, carmustine, etoposide, cytarabine, melphalan/cyclophosphamide; ANC, absolute neutrophil count; CMV, cytomegalovirus; VOD, veno-occlusive disease; TRM, transplant-related mortality. *Statistical significance *P* < 0.05.

**Supplementary Figure 1.** **Progression-free survival (PFS) and overall survival (OS) in patients after transplantation within the GBM/GBC and BEAM/BEAC groups for each major disease subtype in the entire cohort.** **A–B.** DLBCL; **C–D.** PTCL; **E–F.** MCL. GBM/GBC, gemcitabine, busulfan, melphalan/cyclophosphamide; GBC, gemcitabine, busulfan, cyclophosphamide; BEAM/BEAC, carmustine, etoposide, cytarabine, melphalan/cyclophosphamide; DLBCL, diffuse large B-cell lymphoma; PTCL, peripheral T-cell lymphoma; MCL, mantle cell lymphoma.


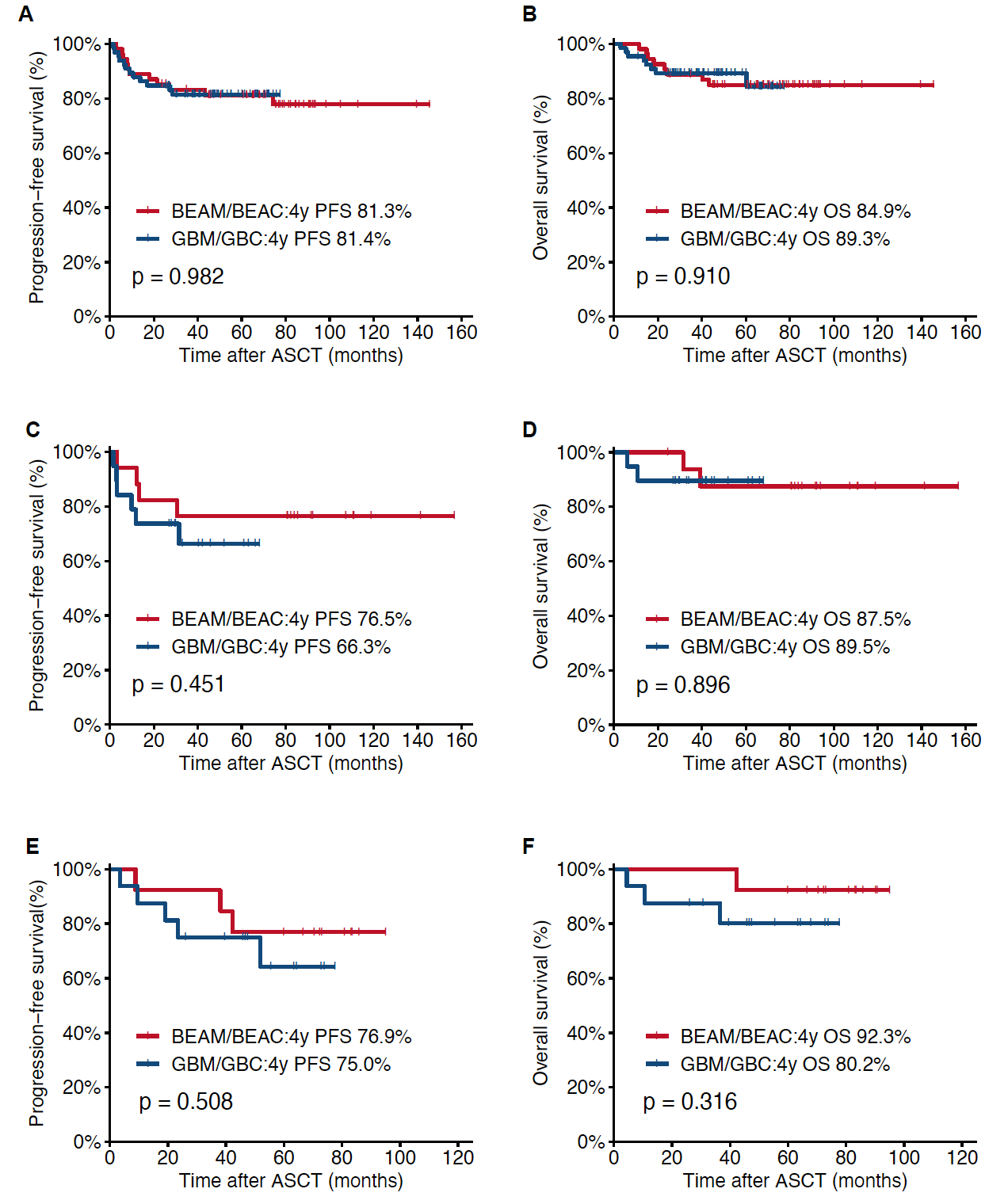


**Supplementary Figure 2. Progression-free survival (PFS) and overall survival (OS) in patients with different prior lines of therapy across the entire cohort. A.** PFS comparison between patients in the first-line treatment group and those in the non-first-line treatment group; **B.** OS comparison between patients in the first-line treatment group and those in the non-first-line treatment group.


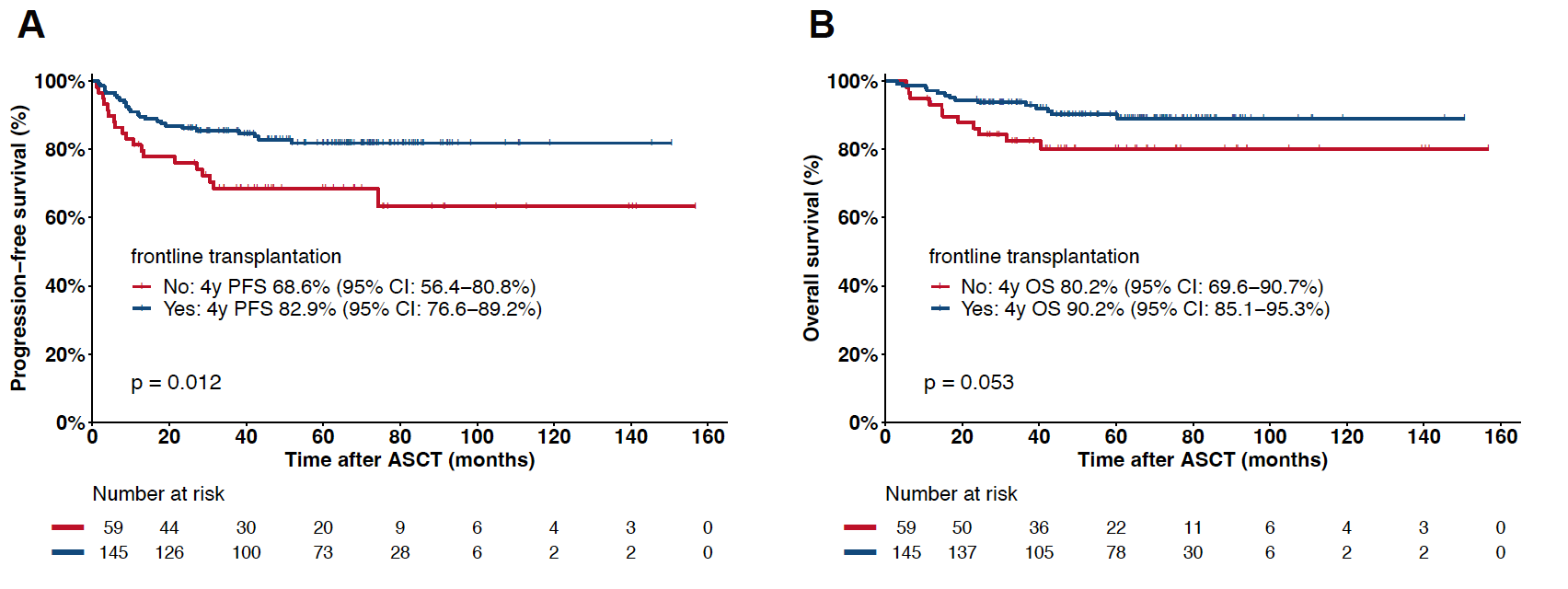


**Supplementary Figure 3. Forest plot of subgroup analysis on progression-free survival (PFS) and overall survival (OS) for the entire cohort by conditioning regimens.** GBM/GBC, gemcitabine, busulfan, melphalan/cyclophosphamide; GBC, gemcitabine, busulfan, cyclophosphamide; BEAM/BEAC, carmustine, etoposide, cytarabine, melphalan/cyclophosphamide; HR: hazard ratio.


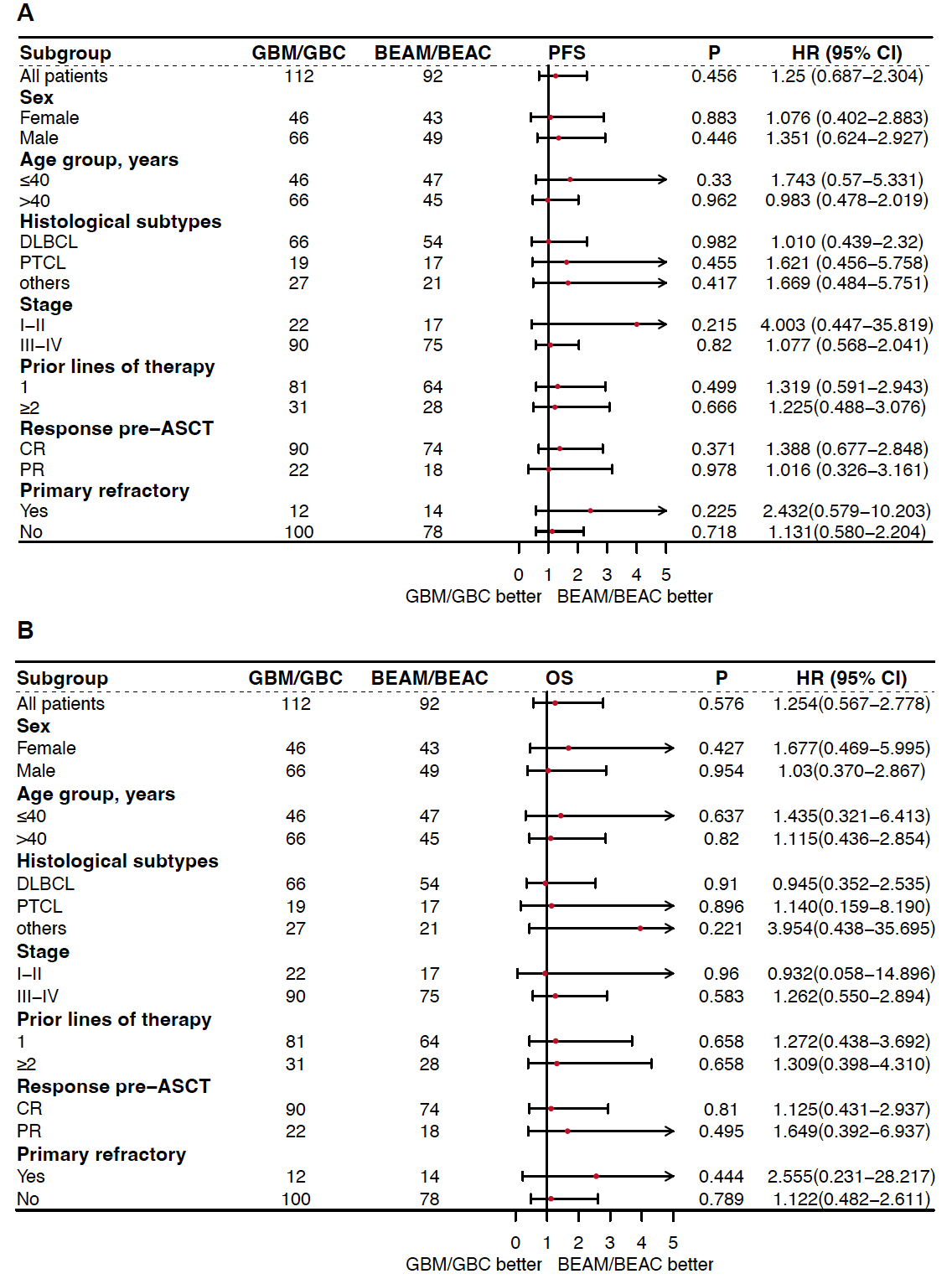

Supplement: Supplementary file 1 — Data S1: Supporting information. [file CAM4-13-e6965-s001.docx]
